# An FDA Analysis of Inspected Entities After Receiving Official Action Indicated Letters for Good Clinical Practice Violations

**DOI:** 10.1007/s43441-021-00267-y

**Published:** 2021-06-08

**Authors:** Miah Jung, Rachelle M. Swann, Michelle S. Anantha, Faranak Jamali

**Affiliations:** Compliance Enforcement Branch, Division of Enforcement and Postmarketing Safety, Office of Scientific Investigations, Office of Compliance, Center for Drug Evaluation and Research, U.S. Food and Drug Administration, 10903 New Hampshire Avenue, Silver Spring, MD 20993 USA

**Keywords:** Bioresearch Monitoring (BIMO), Clinical investigator, Form FDA 483, Good Clinical Practice compliance, Inspection, Sponsor

## Abstract

**Background:**

Limited research has been conducted to examine whether clinical investigators (CIs), sponsors (SPs), contract research organizations (CROs), and sponsor-investigators (SIs) continue conducting clinical trials following issuance of FDA Official Action Indicated (OAI) letters. FDA issues OAI letters for significant regulatory violations. The objective of this study was to evaluate the status of inspected entities who received OAI letters in the conduct of Center for Drug Evaluation and Research (CDER)-regulated clinical trials (CRCTs).

**Methods:**

This cross-sectional study included an analysis of inspectional data from CDER’s Good Clinical Practice (GCP) inspections for OAI letters issued from October 1, 2010, to September 30, 2015, with an in-depth analysis of post-OAI status of inspected entities, including OAI follow-up inspections.

**Results:**

Of the 2248 GCP letters issued during this period, 104 (4.6%) OAI letters were sent: 95 (4.2%) to CIs (91% of OAIs), 7 (0.3%) to SPs (7% of OAIs), and 2 (0.08%) to SIs (2% of OAIs). Majority of OAI letters were issued as a result of a for-cause inspection. Five CIs were excluded from analysis. No OAI letters were sent to CROs. Only 30% of CIs (27 out of 90) continued to conduct CRCTs. OAI follow-up inspections were completed for these CIs resulting in 16 No Action Indicated (NAI), 11 Voluntary Action Indicated (VAI), and no OAI letters. Majority (64%) of the VAI letters noted repeated but not significant violations.

**Conclusions:**

Majority (70%) of CIs who received an OAI letter were no longer conducting CRCTs at the time of follow-up. Of the 27 CIs continuing CRCTs, 16 (59%) OAI follow-up inspections resulted in NAI classifications and 11 (41%) in VAI.

## Introduction

Little research has been conducted to understand the impact of FDA post-inspectional correspondence on CIs, SPs, CROs, and SIs, together addressed as IEs, conducting CRCTs. Although previous research [1] suggests a high turnover rate for CIs conducting CRCTs, limited research has been performed to examine whether IEs continue conducting CRCTs after they receive an FDA OAI letter.

FDA monitors all aspects of the conduct and reporting of FDA-regulated research through BIMO inspections. These inspections are conducted to verify the accuracy and reliability of clinical trial data, ensure protection of subjects’ rights, safety, and welfare, and assess compliance with FDA regulations [2–5]. FDA regulations relevant to CRCTs are found under 21 CFR. For example, 21 CFR Part 50: Protection of Human Subjects provides requirements for informed consent and Part 312: IND provides requirements for the use of investigational new drugs in clinical studies, including the general responsibilities of sponsors and investigators.

Based on the inspectional findings, inspections are classified as follows: NAI, VAI, or OAI. NAI indicates that no violations of FDA regulatory requirements were found. VAI indicates regulatory violations were found, but the violations are not significant enough to require action by the FDA. An OAI classification indicates regulatory violations were identified that significantly impact subject rights, safety, and welfare, and/or significantly compromise data reliability [4, 5]. When an OAI determination is made, FDA issues one of the following letter types: UL, WL, or NIDPOE letter (only for CIs and SIs) [4, 5].

A WL may be issued if the FDA believes that the regulatory violations can be corrected through specific actions and that it is highly likely that adherence to a corrective action plan would prevent similar or other violations in the future. An UL cites violations that do not meet the threshold of regulatory significance for a WL. NIDPOE letters are issued when the CI or SI has repeatedly or deliberately failed to comply with FDA regulations, or has repeatedly or deliberately submitted to FDA or to the sponsor false information in any required report. A NIDPOE letter informs the recipient that FDA is initiating administrative proceedings to determine whether the CI or SI should be disqualified from receiving investigational products pursuant to FDA’s regulation [4, 6, 7].

FDA may continue to monitor IEs following an inspection classified OAI through follow-up inspections or investigations. The decision on whether to conduct an inspection or investigation is based on a thorough post-OAI review of internal and external resources (e.g., FDA databases and extensive online search, incoming reports, and complaints). When search results indicate IEs continue to conduct CRCTs, FDA conducts follow-up inspections to verify whether appropriate corrective actions have been adequately implemented and to ensure that the violations noted in the OAI letter were not repeated in any studies initiated or ongoing after the letter date. If follow-up inspections reveal repeated and/or new significant regulatory violations, FDA may take further action based on the significance, severity, and pattern of the violations.

When search results indicate IEs did not continue to conduct CRCTs, FDA performs investigations (i.e., information-gathering activities) to confirm their post-OAI status (i.e., continuing or not continuing to conduct CRCTs).

## Materials and Methods

In this cross-sectional study, we extracted data from FDA internal databases^[^1^]^ and examined CDER’s records on GCP inspections, including Forms FDA 483 (Inspectional Observations), EIRs and supporting evidence, and the IEs’ written responses to Forms FDA 483 and the resulting OAI letters, and used descriptive analyses to determine the status of the IEs that received OAI letters from October 1, 2010, to September 30, 2015.

Overall inspection data were categorized by the IE type (i.e., CI, SP, CRO, SI) and inspection classification (i.e., NAI, VAI, OAI). OAI inspections were subcategorized by letter type (i.e., UL, WL, NIDPOE letter), geographic location (i.e., domestic (within the United States), foreign), and reason for original inspection (i.e., pre-approval^[^2^]^, for-cause^[^3^]^, or both).

For this study, regulatory violations were grouped into 10 violation themes (Table 1) based on the type of violation cited. In general, the violation themes reflect one or more regulatory citations from 21 CFR.

**Table 1:** Violation Themes

Violation theme	Description	21 CFR section
*Procedures* (CI or SI)	Nonadherence to investigational plan, protocol, investigator statement	312.60
*Protection* (CI or SI)	Failure to protect the rights, safety, and welfare of subjects	312.60
*Oversight* (CI or SI)	Failure to personally conduct or supervise clinical investigations	312.60
*Records* (CI, SP, SI)	Recordkeeping and/or retention violations; failure to provide FDA access to records	312.62, 312.68, 312.57
*Consent* (CI or SI)	Informed consent violations	312.60, 50.20, 50.25, 50.27, 56
*Approval* (CI or SI)	Failure to assure Institutional Review Board (IRB) review, failure to report to IRB changes and unanticipated problems	312.66
*Falsification* (CI or SI)	Repeated or deliberate submission of false information in any required report to FDA or sponsor	312.70(a)
*Controlled Substances* (CI or SI)	Violations in handling of controlled substances	312.69
*Monitoring* (SP or SI)	Noncompliance with general responsibilities; failure to monitor progress of clinical investigations	312.50, 312.56(a)
*IND/NDA* (SP or SI)	Noncompliance with requirements of Investigational New Drug Application (IND) and New Drug Application (NDA)	312.2(a), 312.20(a) and (b), 312.40(a) and (b), 314, 314.50(d)(5)(iv)

With regard to OAI follow-up inspections, we reviewed data for those who continued to conduct CRCTs after receiving an OAI letter. Regulatory violations from the OAI follow-up inspections were grouped into the predefined violation themes and compared with those cited on the original OAI letters.

Violation themes found during the review of OAI follow-up inspections were categorized either as 1) repeated (i.e., recurrence of previously cited regulatory violation); 2) new (i.e., regulatory violation not previously cited); or 3) both repeated and new.

Repeated violations of *Procedures* were further evaluated and categorized as either the same or different subtype. Repeated violations of the same subtype included those noted in both the original OAI and follow-up letters (e.g., enrolling ineligible subjects noted in both letters). Repeated violations of a different subtype included those where the violation differed between the original OAI and follow-up letters (e.g., enrolling ineligible subjects noted in the original OAI letter and failure to perform protocol-required assessments in the follow-up letter).

When the information was available, IEs that were not conducting CRCTs were categorized as retired, resigned, disqualified, deceased, or assumed a non-IE role since the issuance of the original OAI letter.

The analyses for this retrospective study are descriptive and exploratory. Inspectional data were summarized using frequencies and percent for categorical variables. Fisher’s exact test was used to analyze categorical associations.

## Results

## Post-inspectional Classification Analysis

During the study period, CDER’s OSI issued post-inspectional letters for a total of 2248 inspections. These included NAI, VAI, and OAI letters, to CIs, SPs, SIs, and CROs. A majority were issued following inspections conducted domestically (*n* = 1607, 71%) and most were issued for inspections conducted as part of FDA’s marketing application approval process to assess the reliability of submitted data (*n* = 1803, 80%).

**Fig. 1:**
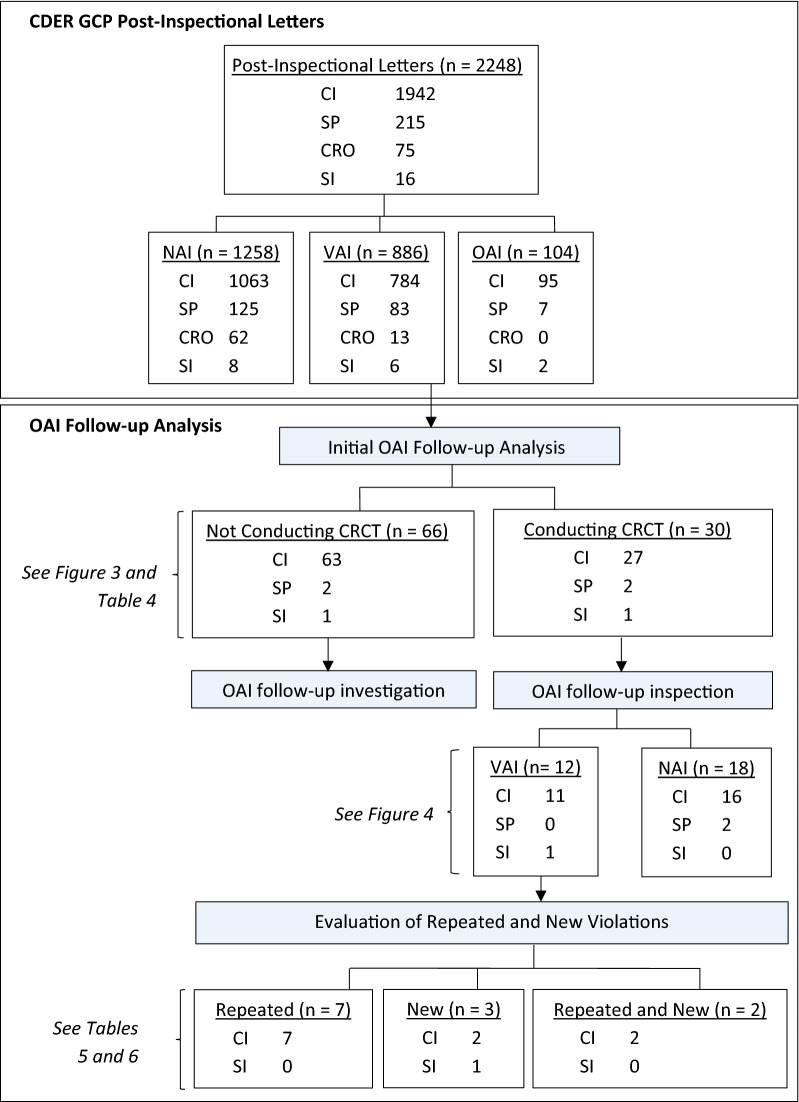
Flowchart Outlining the Selection and Categorization of Inspectional Data. Data cut-off was November 28, 2018, and therefore, five CIs and three SPs, whose statuses were unconfirmed by follow-up inspection or were undergoing evaluation by this date, were excluded from OAI follow-up analysis. See OAI Follow-Up Evaluations—Status of Inspected Entities.

A majority of the inspections were for CIs (*n* = 1942, 86%), followed by SPs (*n* = 215, 10%), CROs (*n* = 75, 3%), and SIs (*n* = 16, 1%). NAI letters (*n* = 1258, 56%) constituted the majority of post-inspectional correspondences for all IEs, followed by VAI letters (*n* = 886, 39%), then OAI letters (*n* = 104, 5%) (Fig. 1).

## Analysis of CDER Post-Inspectional OAI Letters

**Fig. 2:**
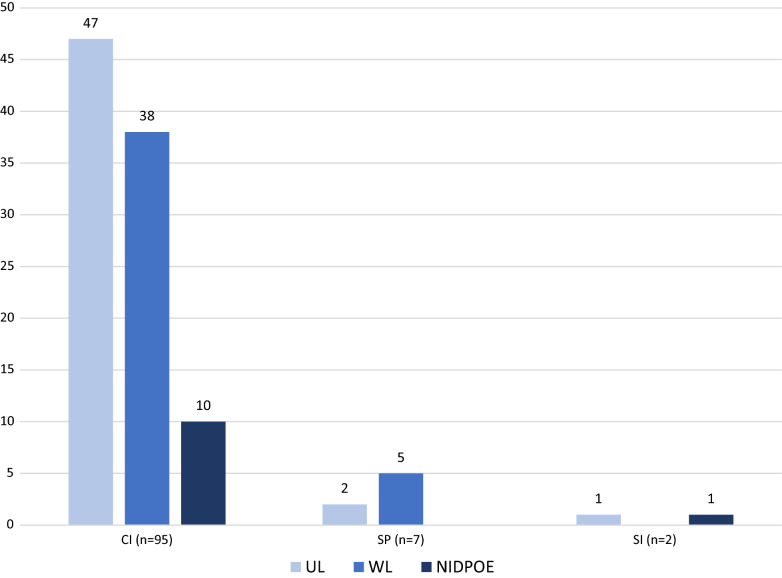
FDA CDER Post-Inspectional OAI Letters by OAI Letter Type

During the study period, OSI issued 104 GCP-related post-inspectional OAI letters to CIs, SPs, and SIs (Fig. 2). The majority of OAI letters, 91% (95 out of 104), were issued to CIs, including 47 ULs, 38 WLs, and 10 NIDPOE letters. SPs received seven OAI letters including two ULs and five WLs, whereas SIs received two OAI letters including one UL and one NIDPOE letter. No OAI letters were issued to CROs during this timeframe; therefore, CROs were not included as part of this CDER post-inspectional OAI letter analysis.

Most of the inspections resulting in an OAI classification were conducted domestically. In addition, the majority of OAI letters were issued as a result of a for-cause inspection (Table 2).

**Table 2:** CDER Post-Inspectional OAI Letters from October 2010 to September 2015

Characteristics	CI (*n* = 95)	SP (*n* = 7)	SI (*n* = 2)	All IEs (*n* = 104)
*Geographic location, No.* (%)				
Domestic	91 (96)	6 (86)	2 (100)	99 (95)
Foreign	4 (4)	1 (14)	0 (0)	5 (5)
*Reason for inspection, No.* (%)				
Pre-approval	22 (23)	2 (29)	0 (0)	24 (23)
For-cause	73 (77)	5 (71)	2 (1)	80 (77)

A total of 224 citations over 10 violation themes were included in OAI letters (Table 3). Most OAI letters cited one violation theme (range 1–6). The mean number of violation themes cited per OAI letter was 2 (SD = 1). Majority of the CI violations were related to either *Procedures* (*n* = 73, 36%) or *Records* (*n* = 68, 33%), followed by *Consent* (*n* = 16, 8%) and *Approval* (*n* = 15, 7%). The most commonly cited violation theme in SP OAI letters was related to *Monitoring* (*n* = 7, 58%), followed by *IND/NDA* (*n* = 4, 33%).

**Table 3:** Number of Citations per Violation Theme Across OAI Letters

Violation Theme, No. (%)	CI (*n* = 206)	SP (*n* = 12)	SI (*n* = 6)	All IEs (*n* = 224)
Procedures	73 (36)	0	1 (17)	74 (33)
Records	68 (33)	1 (8)	2 (33)	71 (32)
Consent	16 (8)	0	0	16 (7)
Approval	15 (7)	0	1 (17)	16 (7)
Oversight	14 (7)	0	0	14 (6)
Falsification	12 (6)	0	0	12 (5)
Protection	7 (3)	0	0	7 (3)
Controlled substances	1 (< 1)	0	0	1 (< 1)
Monitoring	0	7 (58)	0	7 (3)
IND/NDA	0	4 (33)	2 (33)	6 (3)

## OAI Follow-Up Analysis: Status of Inspected Entities

OAI follow-up analysis was conducted to evaluate the percentage of IEs that continued to conduct CRCTs after receiving OAI letters. Based on this analysis, post-OAI status was classified into two main categories: (1) IEs not conducting CRCTs, or (2) IEs conducting CRCTs. The first category included IEs that were retired, resigned, disqualified, and deceased, or no information on future plans was available since the issuance of the OAI letter. The second category included IEs continuing to conduct CRCTs. For these IEs, an analysis of OAI follow-up inspections was conducted to evaluate their conduct of new or ongoing clinical studies initiated or continued after issuance of the original OAI letter. Data cut-off for the follow-up analysis was November 28, 2018. As such, five CIs and three SPs, whose statuses were unconfirmed by follow-up inspection or were undergoing assessment by this date, were excluded from the analysis (Fig. 1).

**Fig. 3:**
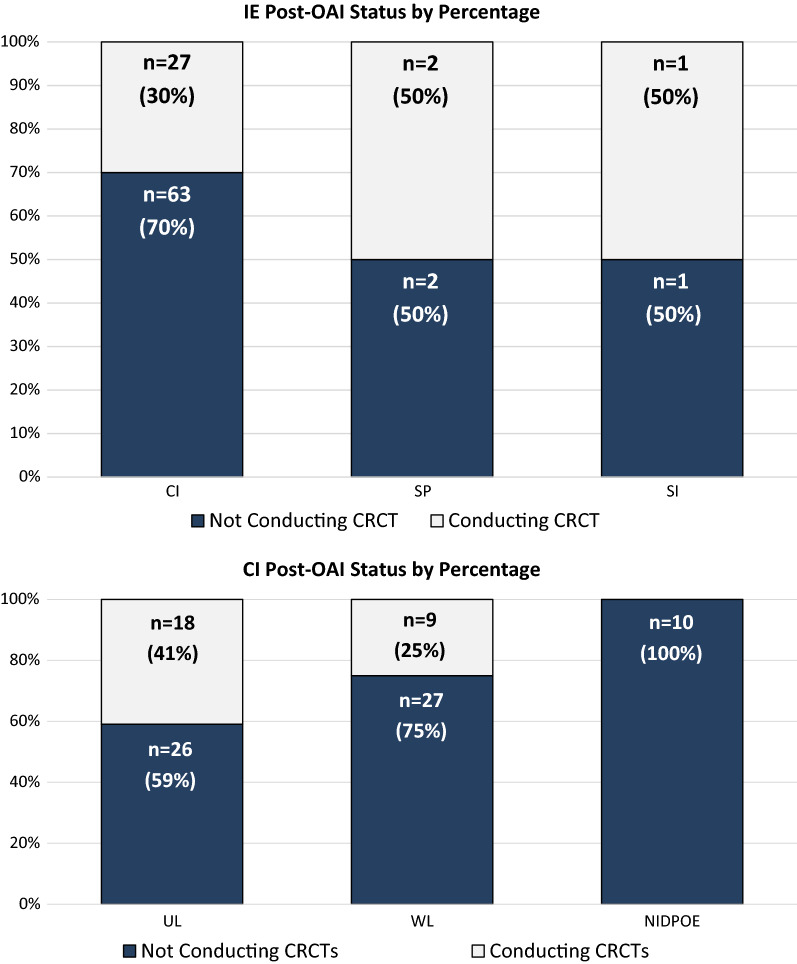
Post-OAI Status by Percentage

Of the 96 IEs, 90 CIs, 4 SPs, and 2 SIs were included in the OAI follow-up analysis. Most CIs (*n* = 63, 70%) did not continue to conduct CRCTs after they received an OAI letter, whereas for SPs and SIs, there was an even split between those that were not conducting CRCTs versus those conducting CRCTs (Fig. 3).

### Clinical Investigator Post-OAI Status

As mentioned earlier, most CIs (*n* = 63, 70%) did not continue to conduct CRCTs after they received an OAI letter (Fig. 3). The original OAI letter type for 26 of these 63 CIs was an UL, for 27 a WL, and for 10 a NIDPOE letter (of which, eight were ultimately disqualified). Among the 27 CIs who continued to conduct CRCTs, 18 had received an UL as the original OAI letter, and nine had received a WL.

The percentage of CIs who did not continue CRCTs after receiving a WL (27 out of 36, 75%) was higher than the percentage of those who did not continue CRCTs after receiving an UL (26 out of 44, 59%); however, no statistically significant difference was detected (p = 0.16 based on Fisher’s exact test, Fig. 3).

Among the 63 CIs who did not continue to conduct CRCTs, 17 CIs were retired or resigned; eight were disqualified; two CIs assumed a non-IE role (i.e., became involved as a sub-investigator) and one was deceased. For the remaining 35 CIs that were not conducting CRCTs, the CIs did not provide information about their future plans to conduct clinical research. See Table 4 for additional breakdown on CIs’ status post-OAI.

**Table 4:** Status Breakdown of CIs Not Conducting CRCTs

Categories, No. (%)	UL	WL	NIDPOE	All CIs
No future plans specified	16 (61)	17 (63)	2 (20)	35 (56)
Retired/Resigned	7 (27)	10 (37)	0 (0)	17 (27)
Disqualified	0 (0)	0 (0)	8 (80)	8 (13)
Assumed non-IE role	2 (8)	0 (0)	0 (0)	2 (3)
Deceased	1 (4)	0 (0)	0 (0)	1 (2)
Total	26	27	10	63

### Sponsor and Sponsor-Investigator Post-OAI Status

OAI follow-up analysis revealed that two of the four SPs continued conducting CRCTs after issuance of the OAI letter, while the remaining two SPs were not conducting CRCTs.

Two OAI letters were issued to SIs during the study period. The SI who received a NIDPOE letter was ultimately disqualified. The SI who received the UL continued to conduct CRCTs.

## Results of OAI Follow-Up Inspections

OAI follow-up inspections are conducted for IEs that continue to conduct CRCTs to determine whether corrective actions are implemented to ensure that the findings noted in the OAI letter are not repeated in any CRCTs initiated or ongoing after issuance of the OAI letter.

A total of 30 OAI follow-up inspections (27 CIs, two SPs, one SI) were conducted of the 96 IEs included in the analysis to evaluate the IE’s conduct of new or ongoing clinical studies that were initiated or continuing after issuance of the original OAI letter.

**Fig. 4:**
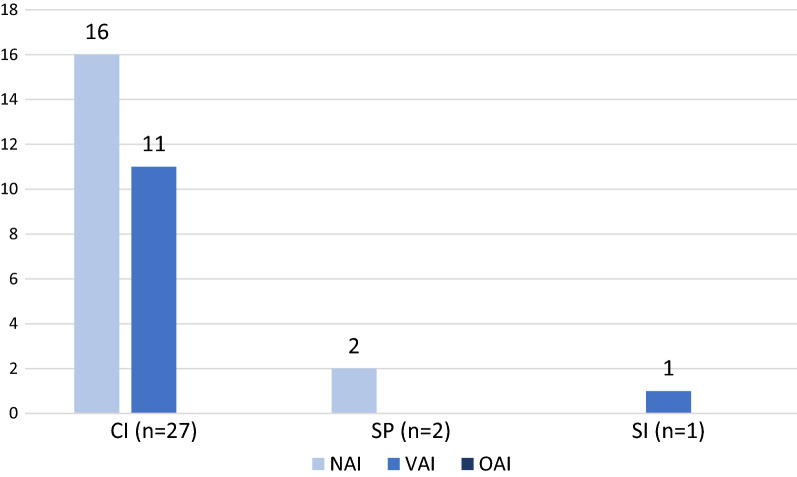
OAI Follow-Up Inspections

For CIs, 16 out of the 27 (59%) OAI follow-up inspections resulted in an NAI letter, and 11 (41%) resulted in a VAI letter. Of the two OAI follow-up inspections of SPs, both resulted in NAI letters. The single SI OAI follow-up inspection resulted in a VAI letter. No OAI follow-up inspections resulted in an OAI letter. See Fig. 4 for further details.

Of the nine CI follow-up inspections that were conducted following issuance of a WL, the majority resulted in a VAI letter (*n* = 6, 67%) as compared to NAI (*n* = 3, 33%). In contrast, among the 18 CI follow-up inspections that were conducted following issuance of an UL, most resulted in an NAI letter (*n* = 13, 72%) as compared to VAI (*n* = 5, 28%). No statistically significant relationship between the original OAI letter types and OAI follow-up results was detected (p = 0.10).

## OAI Follow-Up Inspections Resulting in New, Repeated, or Both Violations

The violations observed during the OAI follow-up inspections were assessed to determine whether they reflected violations cited in the original OAI letter.

**Fig. 5:**
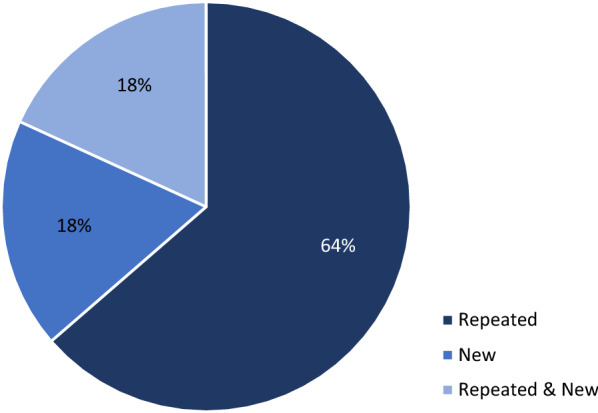
Pattern of Regulatory Violations of OAI Follow-Up CI Inspections Resulting in VAI (*n* = 11)

As noted earlier, 11 VAI letters were issued to CIs and one to an SI as a result of OAI follow-up inspections. Nine of these VAI letters to CIs noted repeated violation themes, whereas two noted new violation themes (Fig. 5). Of the nine VAI letters with repeated violation themes, two had repeated and new violation themes (Fig. 5 and Table 5) and seven had only repeated violation themes.

**Table 5:** OAI Follow-Up Inspections Resulting in VAI with Repeated and/or New Violations by Original OAI Letter Type

Categories by IE type		UL (*n* = 6)	WL (*n* = 6)	All OAI (*n* = 12)
CI, No. (%)		(*n* = 5)	(*n* = 6)	(*n* = 11)
Repeated	Same subtype	3 (60)	2 (33)	5 (46)
	Different subtype	0 (0)	2 (33)	2 (18)
New		2 (40)	0 (0)	2 (18)
Repeated and New		0 (0)	2 (33) ^a^	2 (18)
SI, No. (%)		(*n* = 1)	(*n* = 0)	(*n* = 1)
New		1 (100)	0 (0)	1 (100)

^a^Repeated violations were of same subtype

As presented in Table 3, most CI violations cited in OAI letters were related to *Procedures*. Similarly, most VAI letters (*n* = 7) issued after OAI follow-up inspection noted repeated *Procedures* violations (Table 6). Five of these noted the same subtype. Although repeated violations, these violations did not significantly impact data integrity or subject safety and therefore did not warrant further FDA action.

**Table 6:** Original and OAI Follow-Up Violation Themes for IEs who received a VAI letter following OAI follow-up inspection (CI: *n* = 11; SI: *n* = 1)

IE#	Original OAI Inspection							OAI Follow-up Inspection													
	Original Violation Theme							Repeat Violation Theme							New Violation Theme						
	*Procedures*	*Protection*	*Records*	*Consent*	*Approval*	*Falsification*	*IND/NDA*	*Procedures*	*Protection*	*Records*	*Consent*	*Approval*	*Falsification*	*IND/NDA*	*Procedures*	*Protection*	*Records*	*Consent*	*Approval*	*Falsification*	*IND/NDA*
CI# 1			X																X		
CI# 2						X									X		X				
CI# 3	X	X	X							X								X			
CI# 4	X		X					X^S^											X		
CI# 5	X							X^S^													
CI# 6	X							X^S^													
CI# 7	X							X^D^													
CI# 8	X							X^D^													
CI# 9	X		X	X				X^S^		X	X										
CI# 10	X		X	X				X^S^		X											
CI# 11	X		X	X						X											
SI# 1	X						X										X				

^S^Repeated violation with same subtype

^D^Repeated violation with different subtype

For the one OAI follow-up inspection of an SI, the VAI letter noted a new violation that was different from the one cited in the original OAI letter. The original OAI letter cited *Procedures* and *IND/NDA* deficiencies, whereas the violations observed during OAI follow-up inspection were related to *Records* violations (Table 6).

## Discussion

Our study highlights trends in post-inspectional status of IEs who received OAI letters. First, we found that majority (70%) of CIs who received OAI letters did not continue to conduct CRCTs. Of these, a higher percentage were CIs who received WLs rather than those who received ULs. However, there was no significant statistical difference between these two groups. Post-OAI discontinuation might have been influenced by other contributing factors.

Second, our findings showed that for those IEs who continued to conduct CRCTs, most OAI follow-up inspections resulted in NAI classifications, which demonstrated that no objectionable conditions or practices were observed at the time of follow-up inspection. This suggests that corrective and preventive actions were instituted following the OAI letter. However, inspections, including OAI follow-up inspections, are only a snapshot of an IE’s compliance during a specific timeframe and do not necessarily represent sustained compliance.

Third, those OAI follow-up inspections which resulted in a VAI classification demonstrated that objectionable conditions or practices were found but did not meet the threshold for an OAI classification. For these VAI classifications, most of the violations were repeated, and *Procedures* was the most commonly repeated violation theme during follow-up inspections of CIs. This highlights the need for further improvement in CIs’ conduct of CRCTs in accordance with the investigational plan, protocol, and investigator statement.

A limitation of this study is the brief evaluated timeframe of five years. The small number of OAI letters issued to SPs and SIs during this timeframe limited our ability to draw any definitive conclusions about these IE types. To better understand any trends in post-OAI continuation or discontinuation of CRCTs in all IE types, we suggest data collection over a longer timeframe. Consideration may be given to updating this manuscript in several years’ time.

Furthermore, this study looked only at the post-inspectional status of IEs who received an OAI letter. Additional research assessing the post-inspectional status may help inform whether inspection classification influences IEs’ decision to continue clinical research.

Lastly, we emphasize the importance of conducting high-quality clinical trials and adherence to GCP regulations for the protection of subjects’ rights, safety, and welfare, and ensuring data reliability.

## Conclusions

In this study, we performed OAI follow-up analysis to evaluate the percentage of IEs that continued to conduct CRCTs after receiving OAI letters and performed descriptive analyses of post-OAI status.

The majority of OAI letters were issued as a result of for-cause inspections to CIs. These CIs were located predominantly in domestic sites. The most common violation theme in these original OAI letters was *Procedures*, relating to nonadherence to the investigational plan, protocol, or investigator statement.

Notably, over two-thirds of CIs (63 out of 90, 70%) did not continue to conduct CRCTs after receiving an OAI letter. For those CIs who continued to conduct CRCTs, most of the OAI follow-up inspections resulted in NAI classification and the remaining inspections resulted in a VAI classification. Of the VAI letters issued, the majority noted repeated violations that did not significantly impact data integrity or subject safety. Most repeated regulatory violations were related to *Procedures*. No OAI follow-up inspections resulted in OAI letters.

## Abbreviations

21 CFR: Title 21 of United States Code of Federal Regulations
BIMO: Bioresearch Monitoring
CDER: Center for Drug Evaluation and Research
CFR: Code of Federal Regulations
CI: Clinical Investigator
CRCT: CDER-Regulated Clinical Trial
CRO: Contract Research Organization
EIR: Establishment Inspection Report
FDA: United States Food and Drug Administration
GCP: Good Clinical Practice
IE: Inspected Entity
IND: Investigational New Drug Application
NAI: No Action Indicated
NDA: New Drug Application
NIDPOE: Notice of Initiation of Disqualification Proceedings and Opportunity to Explain
OAI: Official Action Indicated
OSI: Office of Scientific Investigations
SD: Standard Deviation
SI: Sponsor-Investigator
SP: Sponsor
UL: Untitled Letter
VAI: Voluntary Action Indicated
WL: Warning Letter

## Footnotes

1 Accessed November 28, 2018.

2 Pre-approval inspection is conducted by FDA after a sponsor submits to FDA an application to market a new product.

3 For-cause inspection is conducted by FDA to investigate a specific concern or complaint.
